# Integrating statistical and mechanistic approaches with biotic and environmental variables improves model predictions of the impact of climate and land-use changes on future mosquito-vector abundance, diversity and distributions in Australia

**DOI:** 10.1186/s13071-020-04360-3

**Published:** 2020-09-23

**Authors:** Eugene T. Madzokere, Willow Hallgren, Oz Sahin, Julie A. Webster, Cameron E. Webb, Brendan Mackey, Lara J. Herrero

**Affiliations:** 1 Institute for Glycomics, Griffith University, Gold Coast Campus, Southport, QLD 4215 Australia; 2 Environmental Futures Research Institute, Griffith School of Environment, Gold Coast campus, Griffith University, Gold Coast, QLD 4222 Australia; 3 Griffith Climate Change Response Program, Griffith School of Environment, Gold Coast campus, Griffith University, Gold Coast, QLD 4222 Australia; 4 Cities Research Institute, Gold Coast campus, Griffith University, Gold Coast, QLD 4222 Australia; 5 QIMR Berghofer Medical Research Institute, 300 Herston Road, Herston, QLD 4006 Australia; 6 Department of Medical Entomology, NSW Health Pathology, ICPMR, Westmead Hospital, Westmead, NSW 2145 Australia; 7 Marie Bashir Institute of Infectious Diseases and Biosecurity, University of Sydney, Sydney, NSW 2006 Australia

**Keywords:** Mosquito, Distribution, Climate and land-use change, Integrated modelling

## Abstract

Changes to Australia’s climate and land-use patterns could result in expanded spatial and temporal distributions of endemic mosquito vectors including *Aedes* and *Culex* species that transmit medically important arboviruses. Climate and land-use changes greatly influence the suitability of habitats for mosquitoes and their behaviors such as mating, feeding and oviposition. Changes in these behaviors in turn determine future species-specific mosquito diversity, distribution and abundance. In this review, we discuss climate and land-use change factors that influence shifts in mosquito distribution ranges. We also discuss the predictive and epidemiological merits of incorporating these factors into a novel integrated statistical (SSDM) and mechanistic species distribution modelling (MSDM) framework. One potentially significant merit of integrated modelling is an improvement in the future surveillance and control of medically relevant endemic mosquito vectors such as *Aedes vigilax* and *Culex annulirostris*, implicated in the transmission of many arboviruses such as Ross River virus and Barmah Forest virus, and exotic mosquito vectors such as *Aedes aegypti* and *Aedes albopictus*. We conducted a focused literature search to explore the merits of integrating SSDMs and MSDMs with biotic and environmental variables to better predict the future range of endemic mosquito vectors. We show that an integrated framework utilising both SSDMs and MSDMs can improve future mosquito-vector species distribution projections in Australia. We recommend consideration of climate and environmental change projections in the process of developing land-use plans as this directly impacts mosquito-vector distribution and larvae abundance. We also urge laboratory, field-based researchers and modellers to combine these modelling approaches. Having many different variations of integrated (SDM) modelling frameworks could help to enhance the management of endemic mosquitoes in Australia. Enhanced mosquito management measures could in turn lead to lower arbovirus spread and disease notification rates.
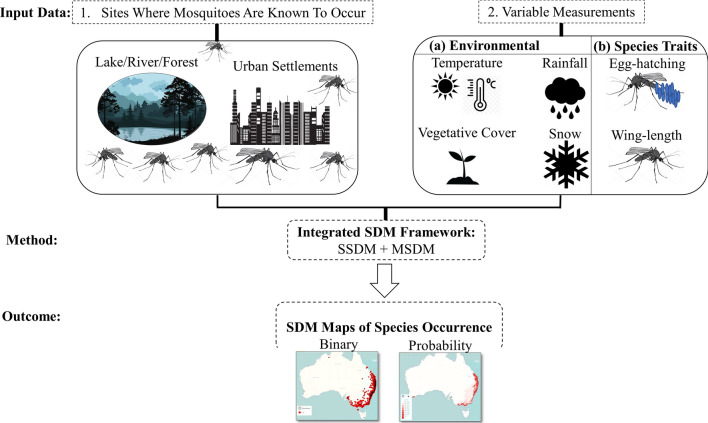

## Background

Australia has a diverse climate range [1], and several of its climatic zones (e.g. tropical, subtropical and temperate) are currently experiencing an increased rate of climatic and land-use changes [2, 3]. These changes may influence mosquito behaviors, such as mating, feeding and oviposition, resulting in an increase in the public health risks associated with mosquitoes in some climatic zones. This development is threatening to overturn the gains of previously effective mosquito management programmes in some regions of Australia [4, 5].

In addition to a diverse climate range, Australia also supports a diverse and, occasionally, abundant mosquito fauna of more than 300 species [6]. Many of the mosquitoes of pest and public health concern occupy several climatic zones. Mosquitoes belonging to the genera *Aedes* and *Culex* are generally thought to be of greatest importance, but there are species belonging to the genera *Anopheles*, *Coquillettidia*, *Mansonia* and *Verrallina* that hold potential as both nuisance-biting pests and vectors of pathogens, including more than 75 medically important arboviruses in Australia [7–9]. Most of these arboviruses, either current or future threats to Australia, are either alphaviruses (e.g. Barmah Forest (BFV), Ross River (RRV), chikungunya (CHIKV) viruses) or flaviviruses (e.g. dengue (DENV), Murray Valley encephalitis (MVEV), Kunjin (KUNV, a subtype of West Nile virus), Zika (ZIKV), Japanese encephalitis (JEV) viruses) [10, 11]. These viruses can cause mild to severe disease epidemics that are costly to manage, can be potentially seriously debilitating, and may result in human mortality [10, 11]. Although the spread of most of these arboviruses in Australia is amplified locally through mosquito-human and mosquito-animal transmission cycles [12, 13], there is currently no record of local transmission of CHIKV and ZIKV by endemic mosquitoes. These specific viruses have only been reported in infected travelers entering Australia [14, 15], but experimental evidence indicates that some endemic haplotypes of the exotic mosquito, *Aedes aegypti* L., can competently transmit them, along with DENV, although this mosquito is currently limited in distribution to central and far north Queensland [16–18]. There are more widespread medically important mosquitoes in Australia including *Aedes vigilax* (Skuse) and *Culex annulirostris* (Skuse) that have the capacity to transmit other arboviruses (e.g. BFV, RRV, MVEV and KUNV) and may place a high burden on the public health system. As a consequence, several of these locally transmitted arboviruses have been listed as significant public health threats in Australia and the diseases they cause are listed as notifiable diseases under federal legislation [11].

In this review, we assess mosquito dispersal, mosquito abundance, and drivers of abundance, diversity and the capacity of integrated species dissemination models to project the most likely future habitat of medically relevant endemic and exotic mosquito vectors under current and future environmental change scenarios.

## Search strategy and selection criteria

We searched Web of Knowledge^TM^ Thomson Reuters, PubMed, Google Scholar and ScienceDirect for original research articles on mosquito dispersal, mosquito abundance, and drivers of abundance, mosquito diversity and the capacity of integrated species distribution models to project the most likely future habitat of medically relevant endemic and exotic mosquito vectors under current and future environmental change scenarios in Australia. Broad search terms were used to maximise the chances of capturing all relevant research articles. We used combinations of the following keywords to search the literature: ‘mosquito’, ‘species distribution model’, ‘correlative’, ‘machine learning’, ‘mechanistic’, ‘joint’, ‘integrated’, ‘combined’, ‘abundance’, ‘community’, ‘spatial’, ‘predictor’, ‘variable’, ‘structure’, ‘profile’, ‘empirical’, ‘seasonal’, ‘biotic’, ‘abiotic’, ‘environmental’, ‘interaction’, ‘drivers’, ‘dispersal’, ‘diversity’, ‘biology’, ‘physiology’, ‘process’, ‘control’, ‘advantage’, ‘disadvantage’, ‘merit’, ‘suppression’, ‘elimination’, ‘*Aedes vigilax’*, ‘*Aedes aegypti’*, ‘*Aedes albopictus’*, ‘*Culex’*, ‘*Aedes’*, ‘*Anopheles’*, ‘*Culex annulirostris’*, ‘Chikungunya virus’, ‘Dengue virus’, ‘Kunjin virus’, ‘Japanese encephalitis virus’, ‘Murray Valley encephalitis virus’, ‘Zika virus’, ‘Ross River virus’, ‘Ross River fever’, ‘Barmah Forest virus’, ‘Usutu virus’, ‘climate’, ‘land-use’, ‘change’, ‘disturbance’, ‘spread’, ‘scenario’, ‘future’, ‘temperature’, ‘rainfall’, ‘wind’, ‘speed’, ‘humidity’, ‘vegetation’, ‘layer’, ‘tidal’. We searched the title, abstracts and full articles for relevant data. We also searched reference lists of selected papers to identify additional articles. All the retrieved articles were screened by two authors (ETM and CEW) based on inclusion and exclusion criteria (Table 1).

**Table 1:** Article selection criteria

Inclusion criteria	Exclusion criteria
(i) Original research article on species distribution modelling	(i) Original articles and reviews not written in English
(ii) Study undertaken in Australia or relevant to:	(ii) Articles on model performance
‣ Mosquito dispersal	(iii) Articles not undertaken in Australia or relevant to what the review seeks to discuss as indicated in inclusion criteria (ii)
‣ Mosquito abundance	
‣ Mosquito diversity	
‣ Mosquito biology and physiology	
‣ Mosquito assembly process and community structure	(iv) Articles not explaining the role of biotic and environmental predictor variables on species distribution projections
‣ Drivers of mosquito abundance	(v) Articles not focused on mosquito-borne viruses circulating in Australia
‣ Mosquito control, suppression, or elimination	(vi) Articles not discussing endemic or exotic mosquitoes to Australia
‣ Impacts of climate and land-use change on mosquito dispersal	
(iii) Study conducted in English	
(iv) Articles explaining the role of biotic and environmental factors on species distribution and abundance	
(v) Original articles on arboviruses such as BFV, CHIKV, DENV, JEV, KUNV, MVEV, RRV and ZIKV, spread by mosquitoes endemic and/or exotic to Australia	
(vi) Articles on endemic or exotic mosquitoes to Australia	

## The mechanisms of mosquito dispersal behavior

Mosquito dispersal behavior is a critical factor in understanding the role of individual mosquito species in disease outbreaks. Notwithstanding the facilitated movement of mosquitoes in the transport of human belongings, natural dispersal may play an important role in the future distribution of mosquitoes, but is unlikely to lead to a significant change in the future range of endemic or exotic mosquito vectors. Instead, future climate change may more strongly influence the seasonality and abundance of endemic mosquitoes rather than the distribution range [3, 13].

The range of mosquito dispersal can be calculated in different ways, including mark-release-recapture experiments and automated mosquito-tracking techniques. Several automated mosquito-tracking techniques have been developed to improve our understanding of mosquito dispersal behavior and a majority of them were recently assessed by Spitzen and Takken [19]. The use of such techniques has revealed that like most insects, all species of mosquito beat their wings at specific frequencies. First, to facilitate flight and allow for dispersal between environments and in search of potential hosts [20], during daylight, at night, or at sunrise and sunset [21]. The second is to generate high frequency tones that modulate courtship between males and females [22]. Automated mosquito-tracking techniques have demonstrated mosquitoes can disperse distances ranging between 50 m and 50 km with flight speed varying by species and environmental conditions [23]. Mark-release-recapture experiments have demonstrated that individual mosquito species, even those within the same genus, vary in their dispersal. Mosquitoes of the genus *Aedes* have been dispersing over distances less than 200 m (e.g. *Ae. aegypti*) [24], but also over 3 km (e.g. *Ae*. *vigilax*) [25], while those belonging to *Culex* and several other genera (e.g. *Culiseta*, *Coquillettidia*, *Mansonia* and *Psorophora*) are capable of withstanding and surviving long distance dispersal [23]. The dispersal ranges of mosquitoes are no doubt instrumental to the scale of impact of medically relevant arboviruses such as RRV and BFV in Australia.

## Factors influencing mosquito dispersal

Research has identified three factors that critically influence the dispersal of a mosquito vector: (i) its physiology, (ii) the type of vegetation communities, and (iii) the conditions in the mosquito’s environment [23]. A change in any or all these factors can impact a mosquito’s capacity to disperse over short or long distances. As an example, changes in mosquito physiology such as an increase in the accumulation of glycogen stores in fibrillar thoracic muscles, can occur when mosquitoes ingest nectar from plants [26, 27]. This provides mosquitoes with energy to fly over long distances. When mosquitoes ingest blood meals from animal hosts their abdomen becomes distended and they become heavier and are more inclined to rest or only fly over short distances [27]. Nectar and blood meals are rich in metabolites such as carbohydrates [28], lipids [29], amino acids such as proline [30, 31], and vitamins, metals and salts [32]. Several studies have shown that when processed, these metabolites generate energy reserves that help the mosquito to physiologically sustain either short or long-distance dispersal [27–32].

Modifying or restoring vegetation in the environment where mosquitoes reside can also impact mosquito spread because vegetation is a source of nutrition and shelter for adult and immature mosquitoes [33]. Changes in vegetative cover can occur naturally, but they are often the outcome of landscape disturbance brought about by land clearance, urbanisation and vector-control initiatives [3, 33]. In Australia, there are four broad categories of vegetation community type: (i) forest; (ii) rainforest; (iii) grassland; and (iv) desert. Most species of endemic mosquito traditionally occupy areas with the first three types of vegetation because these areas are inundated with vegetative cover (e.g. trees, shrubs, grasses) which they can use as food and energy sources, resting, breeding and oviposition sites [3, 33]. However, the diversity of habitats within each of these categories can be significant and for individual mosquito species, there will be a suite of environmental factors that provide ideal conditions conducive for abundant mosquito populations; these can include freshwater, brackishwater or saline environments, as well as those predominantly permanent, semi-permanent, or ephemeral. Therefore, both increasing and decreasing the density of vegetation in an environment can lead to the spread of mosquitoes in search of environments with suitable vegetative cover, especially where these conditions support wildlife representing blood sources for mosquitoes.

Changes in environmental and climatic conditions such as temperature, rainfall, humidity, light intensity, vegetation, and wind speed play a critical role in determining the relative abundance and diversity of mosquito populations and their associations with specific habitat types [3]. For example, temperature plays a key role in determining many aspects of mosquito biology and pathogen transmission [34, 35]. Mosquito dispersal has been shown to optimally occur at temperatures ranging between 15 °C and 32 °C [34] and, in Australia, low to moderately high temperatures have also been associated with high growth and survival rates for both *Ae. aegypti* and *Culex quinquefasciatus* Say [36, 37]. Low to moderately high (between 18 and 24 °C) and extreme (above 35 °C) mosquito larvae rearing temperatures can also aid the transmission of arboviruses by mosquitoes [38, 39]. For example, low to moderately high temperatures have previously been reported to destabilize RNA interference, increasing the lifespan and the capacity of CHIKV to replicate and disseminate in both *Ae. aegypti* and *Aedes albopictus* (Skuse) [40]. Similarly, while temperatures above 35 °C will kill most species of mosquito, they have also been reported to shorten the time it takes for arboviruses to replicate and disseminate to the salivary glands of mosquitoes, accelerating arboviral transmission rates from species such as *Cx. quinquefasciatus* to susceptible hosts [41]. Cooler temperatures (< 18 °C) may selectively promote proliferation of mosquitoes capable of adapting to tree-hole breeding such as *Ae. albopictus* [42]. In addition to temperature, humidity levels in the environment between 30 and 80% have previously been shown to provide optimal conditions for mosquito dispersal [34]. There are also many gaps in current understandings of how mosquitoes interact with the local environment in response to temperature extremes and the exploitation of microclimates may mean that some mosquitoes are far more tolerant and resilient to the potentially adverse impacts of temperature.

Like temperature and humidity, rainfall also plays a critical role in driving mosquito abundance and longevity. Greater than average rainfall has strongly been associated with both high abundance of several *Aedes* and *Culex* mosquito-vector species as well as recurrent cycles of RRV and BFV epidemics in Australia [43, 44]. Future changes in rainfall are likely to substantially influence the abundance and diversity of local mosquito populations, especially in cases where there is a shift in seasonal rainfall patterns [13]. Given the species-specific relationships between mosquitoes and habitat characteristics, such as the permanent or ephemeral nature of waterbodies, increased rainfall is likely to increase the diversity of mosquito species within a local area by creating a wider range of habitats. There is also likely to be greater interaction between freshwater and estuarine wetlands that may further enhance, but in some circumstances reduce, the abundance of suitable habitats for mosquitoes. Flooding too, has been shown to drive increases in mosquitoes and associated mosquito-borne disease risk in south-east Australia [45]. However, complex relationships between the abundance and diversity of mosquito populations and mosquito-borne disease mean that the predictive role of rainfall is not always reliable.

While low wind speeds can reduce mosquito host-seeking dispersal, studies have suggested that high altitude wind dispersal of mosquitoes may extend to hundreds of kilometers and, potentially, introduce pathogens to new regions through long-distance dispersal of infected mosquitoes [46]. This is how mosquitoes carrying JEV from Papua New Guinea are believed to have reached northern Australia resulting in JEV outbreaks that occurred in 1995 and 1998 [47]. Predicted increasing frequency and intensity of tropical cyclones impacting northern Australia [48], may facilitate movement of vectors or pathogens into mainland regions.

Several human activities that alter the ecology of an environment can also influence mosquito spread. These activities include (i) urbanisation, (ii) the application of insecticides (e.g. larvicides, insect growth regulators) and mosquito predators (e.g. birds, fish, bats, *Mesocylclops* copepods or mosquitoes of the genus *Toxorhynchites*) into a larvae and mosquito infested area [49], (iii) construction of water-holding bodies (e.g. dams, canals) and/or wetlands, and (iv) animal husbandry [50]. They stimulate changes in mosquito behavior (i.e. mating, food-, host-, breeding- and oviposition-site seeking) and modify local environmental conditions so as to selectively influence a species capacity to colonise, proliferate and spread in the modified or to another suitable environment.

Fortunately, changes in the environment and climatic conditions in mosquito habitats can also be used to improve our understanding of mosquito dispersal. One way of achieving this is to design models that can account for the impacts of biological, environmental and/or human oriented factors on mosquito dispersal to improve predictions of the most likely future habitats of epidemiologically relevant mosquito-vector species. While there will be a need to fill gaps in the understanding of biology and ecology of many mosquito species, such models have the potential to improve species-specific mosquito surveillance, control and suppression of abundance or elimination in the future.

## The future range and public health threat of exotic mosquito vectors

Two of the most common and widely distributed endemic mosquitoes in Australia are *Cx. annulirostris* and *Ae. vigilax*. Future changes in environmental conditions and climatic variables within local mosquito habitats will likely only influence the seasonality and abundance of both vectors but not significantly their geographical range [3]. Compared to *Cx. annulirostris* and *Ae. vigilax*, exotic mosquitoes such as *Ae. aegypti* and *Ae. albopictus*, but also *Culex gelidus* (Theobald), and *Culex pipiens molestus* Forskal, have a spatially restricted geographical range in Australia [6, 51]. Exotic mosquitoes are also considered potentially high-risk pests that could be highly resistant to locally applied insecticides and may also have a vectorial capacity that exceeds the one exhibited by local mosquitoes. As such exotic mosquitoes, especially *Ae. aegypti* and *Ae. albopictus*, could bring with them to Australia increased risk of local transmission of pathogens such as DENV, JEV, CHIKV, WNV and ZIKV, which can cause more severe, debilitating and life-threatening illness. Preventing incursions of these exotic mosquitoes into Australia from neighboring countries and islands in the Pacific should remain a major public health priority. Recent incursions of *Ae. aegypti* and *Ae. albopictus* into the Northern Territory have been detected and thwarted at Darwin port [52, 53]. Most exotic *Aedes* vector importations at Darwin port have been associated with illegal fishing vessels [54] and more recently cargo vessels [52]. Incursions of foreign *Ae. aegypti* haplotypes have also been reported at Darwin and several other Australian international airports such as Perth, Melbourne, Adelaide, Brisbane and Sydney [55]. As the number of exotic vector detections increases, there is growing concern that, should these exotic vectors evade detection at sea and other ports of entry into Australia and establish on the mainland, they could quickly adapt to local environmental and climatic conditions and spread rapidly to achieve a wide distribution. If this happens, local and state governments could become overwhelmed and struggle to control the new exotic mosquito populations because as things stand, there are not enough resources to control the spread of local mosquitoes and the arboviruses they carry [4, 5]. For this reason, all models predicting the future range of mosquitoes in Australia should be trained and validated to account for the adaptation and dispersal dynamics of exotic vectors as they also have a large public health impact.

## The public health impact of environmental changes projected for Australia

### Climate change projections: Representative Concentration Pathway (RCP) 8.5

For the past decade, carbon dioxide emissions in Australia have mostly tracked the highest Intergovernmental Panel on Climate Change (IPCC) scenario, RCP 8.5 [56]. Therefore, we summarise here the current trends and projected changes in key climatic variables for only this most severe of the four climate change emission scenarios as described in the Fifth Assessment Report of the IPCC [56]. RCPs highlight distinct greenhouse gas, aerosol and land-use change emission scenarios, and RCP 8.5 specifically indicates the highest emission scenario, characterised by increases in emissions leading to a CO_2_ concentration in the atmosphere of approximately 940 parts per million (ppm) by the year 2100 [2]. Under scenario RCP 8.5, an overall increase in mean temperatures (2.8–5.1 °C), and geographically varying alterations in mean wind speed (decreases not expected to exceed 10%), precipitation/rainfall regimes (both excessively low and high rainfall patterns), and humidity (decline in inland regions, and during winter and spring seasons) are projected for Australia by 2030 (2020–2040) and 2100 (2090–2110) compared to 2015 (2005–2025) [2]. For example, more hot days, heat waves, droughts and fewer cold days are projected by 2090 (2080–2100). Due to increasing mean temperatures, areas with the highest probability of experiencing frost annually may no longer do so by 2030 (2020–2040). Coastal areas, suitable habitats for supporting large distributions of *Aedes* and *Culex* mosquito-vector species, might also be frost-free in 2090 (2080–2100) and this could impact the future seasonal distribution of these vectors. Also, the southward movement of three winter atmospheric systems is projected to result in a decline of southwestern Australiaʼs winter rainfall by up to 50% by 2090 (2080–2100). This scenario will likely favour an increase in the spread of saltmarsh mosquitoes such as *Ae. vigilax* in this area that, in combination with the persistence of the southern saltmarsh mosquito, *Aedes camptorhynchus* (Thomson), may increase the pest and public health risks associated with mosquitoes in southern Australia because the eggs of these saltmarsh mosquitoes can survive long periods of dry conditions and hatch in large numbers when they come into contact even will little amounts of rainfall [57]. In addition to changes in climate, sea level rise and concomitant changes in tidal inundation of coastal habitats will also influence the abundance and seasonality of mosquitoes associated with estuarine wetlands.

### Projections for land-use land-cover change: Possible after-effects of the “Developing Northern Australia” Project

The Australian Commonwealth Government released a white paper in 2015 on the “Developing Northern Australia” project [58]. If approved and implemented, this project will reshape mosquito-vector ecologies in northern Australia by significantly modifying land-use practices in traditionally rural, forest covered and human uninhabited areas [58]. Northern Australia’s human population is projected to surpass 2.9 million by 2050, increasing human occupation of lands in close proximity to productive mosquito habitats as well as modification of local mosquito habitats directly and indirectly through expanded human settlements [59].

The process of creating new human settlements often involves forest logging, clearance of large areas of vegetative land cover, damage to swamps and salt-marshes, and the establishment of temporary and/or permanent water-holding bodies (e.g. pools, ponds, dams, canals) [3]. These land-use disturbances can directly and indirectly change the likely exposure of humans to mosquito-borne pathogens due to an increase in mosquito habitats and host seeking behaviour resulting in increased vector and disease spread [50]. Urban development, including the construction of artificial waterbodies, may indirectly expose the community to the pest and public health risks of mosquitoes. Changes in the land-use around urban estuarine wetlands has been demonstrated to influence the abundance and diversity of mosquitoes [60] and increasing urbanisation in coastal regions is predisposing a greater proportion of the community to mosquitoes and mosquito-borne disease [61].

Land-use changes could also lead to the release of large amounts of CO_2_ into the atmosphere leading to an increase in the surface and air-temperature in northern Australia, as has historically been observed to occur in this region as a result of climate change [2]. This could make local habitats more suitable for occupation by large populations of different species of endemic or exotic *Aedes* and *Culex* mosquitoes that thrive in warmer environments [62]. Increased abundance of vector mosquitoes may result directly from changing temperature, temporal rainfall patterns, extreme weather events, and sea level rise. However, changes to storm-water and waste-water management (e.g. constructed wetlands) in association with expanding urban developments may also bring increased, or enhanced, conditions for a suite of vector mosquitoes [63]. The potential for increased exposure to mosquitoes through poorly planned urban developments has been acknowledged in both Australia and North America [61]. Notwithstanding future increases in endemic vector mosquitoes, urbanisation can bring increased risk of introduction and establishment of exotic container-inhabiting mosquitoes not currently present in the region. Combined with expected increases in movement of travellers between northern Australia and countries experiencing endemic activity of mosquito-borne diseases caused by DENV, ZIKV, and CHIKV, the risk of local transmission may increase. This would increase the burden of arboviral disease in northern Australia. One way of averting this is to carefully consider and account for all potentially relevant ecological and epidemiological impacts of the “Developing Northern Australia” project and develop strategies that can be implemented to address these impacts. Otherwise, the new inhabitants of northern Australia will be at a greater risk of exposure to arboviral infections, many of which cause incurable and life-threatening illnesses.

## Managing the spread of mosquito vectors in Australia

There are many factors that complicate efforts to control the abundance and spread of medically relevant mosquito vectors in Australia. These factors include limited access to the financial and operational resources required to effectively manage local mosquito populations as well as an increase in land-use and environmental change. Resources that are often limiting include funding and a suitably qualified workforce to support mosquito surveillance and elimination programmes throughout the country. While some local authorities may secure appropriate funding to undertake active management of local mosquito populations that represent substantial nuisance impacts, exotic mosquitoes that bring with them potentially significant public health risks may be more likely to be perceived as a far more urgent matter requiring response. This has been the case where eradication programmes have been required in response to detection of *Ae. aegypti* in the Northern Territory [64], and credit must be given to the Commonwealth Government of Australia for consistently pledging approximately AU$1 million dollars since 2013 to support mosquito control particularly in the Torres Strait region so as to keep *Ae. albopictus* off the mainland [65, 66]. However, in Queensland and New South Wales, financial constraints continue to limit the ability of authorities to control the spread of endemic mosquitoes as the cost of doing so has been increasing from AU$7 to AU$11 million per year between 1993 and 2004 [4, 5], to above AU$20 million per year by 2014 [67]. Even an *Ae. albopictus* eradication strategy implemented in Brisbane (Queensland) was recently projected to cost around AU$1.2 million [68]. Clearly, more funding is required to recruit, train, equip (with adequate control resources such as traps and insecticide) and transport the workforce to surveillance and elimination sites. More funding is also required to support research aimed at developing biological and chemical controls and determining evidence and sources of resistance to insecticides as has been reported for *Aedes* mosquito vectors in other regions of the world [69]. To improve the control of mosquito spread, impacts of increasing land-use and environmental change must also be carefully studied and addressed. One way of doing so is to account for and address such changes in land development plans. Another approach is to develop models that can account for these changes and predict the most likely suitable habitats of mosquito vectors in the future under a wide range of land-use and environmental change scenarios. Such models can be used to guide and improve mosquito control. Below, we review the classes, differences, advantages and disadvantages of these models. We also highlight how when different types of such models are used in an integrated framework this can improve predictions of the future range of endemic or exotic mosquitoes in Australia.

## Mosquito-vector species distribution models

### Categories and classes of species distribution models (SDM)

The term “SDM” describes a range of empirical models that employ computer algorithms, species occurrence data, climate and a range of potential predictor variables, such as vegetation and land-use, to predict the most suitable sites for mosquitoes to move to, occur and/or persist in [70]. As these models can statistically associate or correlate mosquito-species occurrence data to climatic and land-use variations [70], they are invaluable tools for assessing the adaptive capacity of mosquito vectors under future scenarios. There are two commonly utilised categories of SDMs; the statistical SDMs (SSDMs) and the mechanistic SDMs (MSDMs).

Correlative models are SSDMs that can correlate species occurrence data with environmental predictors. These models are successfully used on large scales in time and space [71], and need to be trained on a subset of the occurrence data and validated to continuously improve prediction outcomes [70]. An overview of several examples of correlative SDMs (i.e. profile-, regression- and machine learning-based) has been given by Peterson et al. [70].

MSDMs are unique in that they simulate the process by which mosquito-vector physiology and behavioral traits evolve in response to climate and land-use changes enabling mosquito vectors to colonise, spread and populate a suitable habitat [70, 72, 73]. SSDMs and MSDMs can be combined or linked in many ways and a few of these combinatorial strategies will be the focus of the section below.

It is worth noting that there is another type of SDM that has recently been developed; Joint SDMs (JSDMs). This form of SDM utilises latent (unobserved) variables, generalised linear regression, and neural network approaches to account for biotic interactions and missing environmental predictors in correlative species distribution models. This helps to analyse the spatio-temporal structure and correlated distribution of multiple species at multiple hierarchical levels [74–76]. JSDMs can identify and quantify the effects of biotic interactions such as predation, competition and mutualism on species distributions [77]. Incorporation and specification of biotic factors in JSDMs improves our understanding of the processes underlying species assembly and lowers bias in the prediction of species community structure [78, 79]. The performance of various presence-absence JSDMs has been assessed at length [74]. JSDMs are considered computationally efficient but memory intensive and poor at evaluating species associations [74, 77], as a result these models will not be discussed further.

### Combining SSDMs and MSDMs

There are currently three possible ways to link or combine SSDM and MSDM approaches in a species distribution modelling framework. The first method involves comparing outputs of both the SSDM and MSDM for the same species. Predictions made by the two SDMs will either concur or not. Model concurrence will indicate greater confidence in the predictions made, as was the case in Strasburg et al. [80] for the gecko *H. binoei*; while model disagreement presents avenues for the development of alternative hypotheses. The second method involves using MSDMs to generate highly proximal geographical outputs on which to base the correlative modelling analyses [81, 82]. This can be achieved by ensuring that the MSDM includes surfaces such as the potential activity time, water loss, energy costs and development time for a given species within a specific terrain and vegetation; these independent variables can improve and make SSDMs more interpretable. The third and last way by which to link SSDMs and MSDMs is by using the MSDM to define the geographical scope of the SSDM. This is usually achieved by first using the MSDM to identify areas on a geographical landscape where the species clearly does not exist. The next step involves tightly focusing the SSDM by placing absence points in such areas or through the exclusion of such areas in the SDM analysis altogether [83].

Combining SSDMs and MSDMs into an integrated modelling framework which utilises climate and land-use change data can reduce uncertainty in and improve predictions of the most likely suitable future habitats and abundances for endemic *Aedes* and *Culex* mosquitoes in Australia and other countries [72, 84–87]. Because combining SSDMs with MSDMs into an integrated modelling framework can have such a considerable effect on the surveillance and control of endemic mosquitoes, we give a detailed account of the contrast between these two types of species distribution modeling approaches and how the differences between them can impact a species projected future distribution.

### Contrasting SSDM and MSDM modeling approaches

There are significant differences between SSDM and MSDM approaches to species distribution modelling. For example, MSDMs can account for a mosquito-vector species responses (both physiological and population) to changes in climatic and land-use variables within the species current habitat [73]. This includes land-use variables such as soil type, soil moisture content, distance to roads and/or waterbodies, altitude, slope, ground coverage, and annual net land-atmosphere carbon flux. Population density can also be considered as a land-use variable. However, the use of MSDMs is often limited by the lack of data on a species response to changing environmental variables. It may be the case that there are substantial gaps in the understanding of individual mosquito species, their habitat associations, and environmental drivers of abundance. Currently gaps remain in our understanding of some endemic mosquito species and their ecological associations with vegetation and water quality requirements of habitats as well as the seasonal rainfall patterns [6]. Temporal distributions of rainfall may influence, both positively or negatively, the abundance and diversity of local mosquitoes associated with certain ecological niches. For example, mosquitoes associated with estuarine saltmarshes and mangroves may respond to above average rainfall differently to mosquitoes associated with coastal swamp forests or inland flood plain habitats [6].

There are also significant differences in SSDM and MSDM model assumptions and these influence species distribution predictions. MSDMs for example, surmise that existing geographical range of a species is a highly informative indicator of its physiological and behavioral responses and tolerance to changing climatic and other environmental variables within a given location or habitat [73]. In contrast to MSDMs, SSDMs are significantly reliant upon empirical associations; they have little to zero mechanistic underpinning and assume that a species’ current distribution gives the best indication of its ecological requirements [73, 88]. The existence of very limited mechanistic conception in SSDMs explains why they do not explicitly identify and represent the processes (physiological and environmental) that limit a given species range [83]. This is the main advantage that MSDMs have over SSDMs. However, a major limitation to the use of MSDMs is their requirement of experimental data on species tolerances and responses to a set of environmental variables in order to generate a species projection [83]. These data are either experimentally difficult to generate and are often not readily available for use in SDM experiments. Regarding mosquitoes, there may be a paucity of data on the biology or ecology of mosquitoes within certain geographical ranges or under specific environment conditions. This may be relevant at the boundary of new geographical regions of a mosquito’s distribution where climatic conditions may become suitable but where vegetation communities in habitats may not similarly have adapted. Since SSDMs do not have this requirement, they have been used much more widely compared to MSDMs to generate species projections [83]. However, this does not mean that SSDMs are significantly better than MSDMs as a surveillance tool, as both types of model can generate congruent and divergent species projections [89, 90]. In fact, generating species projections based on SSDMs alone should be discouraged because these models have overly simplistic assumptions and such exercise may hamper the design of measures to mitigate the spread of medically relevant mosquito vectors in Australia. As an example, several indices (e.g. Breteau and Container) used by SSDMs to generate future species projections have been criticised for failing to accurately quantify the abundance of adult *Ae. aegypti* mosquito vectors in Australia [91]. For this reason, we believe that more realistic and reliable future mosquito-vector species projections can only be obtained with an integrated modelling approach which utilises a combination of SSDMs and MSDMs as well as climate and land-use change data. To improve the modeling of future mosquito species range, such an integrated modelling framework should be trained and validated to fully account for mosquito dispersal dynamics so as to help to create species-specific consensus species distribution maps that can be used to concentrate mosquito-vector surveillance and control in areas where the mosquito range is both currently high and predicted to expand in the future. The advantage of this approach is that it may also assist in minimising the spread of mosquito-borne disease where outcomes can inform local or regional mosquito management or response plans.

### Merits of integrated mosquito-vector species distribution modelling

Several publications have reviewed the advantages and disadvantages of both single and combined SDM approaches [73, 83, 89, 90]. Here, we discuss only the advantages associated with the use of an integrated SDM modelling framework. Two significant advantages to using an integrated modeling framework are the reduction in uncertainty and bias or sources of errors in the generation of species projections [92]. Uncertainty and bias are two significant sources of error in species projections. Uncertainty in MSDM projections can be higher than in SSDM projections because MSDMs account for a greater number of model parameters to better represent all the processes that influence a species capacity to shift ranges [89]. However, both uncertainty and bias in species projections can be significantly reduced through model integration [83, 92]. An integrated modelling framework consisting of a combination of SSDMs and MSDMs utilising climate and land-use change data, is considered to be more scalable, powerful, and capable of generating more realistic consensus species distribution projections than a single-model SDM approach [84, 90, 92]. Realistic projections of future species range shifts are a vital resource in the fight to minimise the spread of medically important mosquito vectors and the arboviruses they carry in Australia. The use of an integrated modelling framework is also advantageous in many other respects. For example, it presents an opportunity to quantify responses of mosquito species that do not yet exist under future climate and land-use change scenarios and such a framework can be trained and validated to consistently generate improved, consensus species distribution projections [92]. Further approaches combining SDMs with specific dispersal abilities and least cost paths can be helpful, too, to inform public health authorities [93, 94].

### Projections generated using integrated mosquito-vector species distribution models

While there are several merits linked to integration of predictions from correlative and mechanistic SDM approaches such as robust forecasting of climate and land-use change impacts on the suitability of a habitat for a given species, SSDMs and MSDMs have only been used together once in an integrated modelling framework in 2014 to project the future distribution of *Ae. albopictus* in Australia under scenario RCP 8.5. In the study where this principle was illustrated in 2014, Hill et al. [85] used *MaxEnt* (a machine learning SDM: an SSDM) with *CLIMEX* (a semi-mechanistic process-based model: an MSDM) in an integrated modelling framework where they linked the models by comparing their output and generated projections suggesting that by 2030 and 2050, *Ae. albopictus* would become widely distributed along the entire north and eastern coast and remain restricted to the coastal fringe; and that from these regions the vector would extend inland into Western Australia, and southward into Southern Australia and Tasmania [84]. These projections also suggested that this vector would predominantly be introduced into Australia from Asia and North America, where it is known to circulate widely [84]. At present monitoring data on Ae. albopictus indicates that this vector is not in Australia but Hill et al. [85] projections suggest it can invade certain areas. Two factors motivated the decision to generate species projections for *Ae*. *albopictus* using an integrated modelling framework. First, next to *Ae. aegypti*, *Ae. albopictus* is globally the second most important vector of DENV in areas of endemicity [95]. Its presence in Australia alongside *Ae. aegypti* would expand the range of DENV, which is currently restricted to central and north Queensland where *Ae. aegypti* is also widely distributed [95]. Over the past decade or so, the abundance of *Ae. aegypti* and incidents of DENV in Cairns and Townsville have significantly reduced due to the success of large-scale deployment of *Wolbachia* (wMel) *Aedes*-infected mosquitoes [96, 97]. Secondly, unlike *Ae. aegypti*, *Ae*. *albopictus* carries with it a much larger epidemiological threat as it can readily adapt to temperate and cold environments such as those prevalent in southern Australia [98–100], and it can also transmit DENV better in areas where the virus does not currently circulate [95]. To prevent the spread of this vector and DENV in this country, species projections generated by Hill et al. [85] have been used to concentrate *Ae. albopictus* control and elimination programmes in areas where the vector is currently widely distributed (i.e. Torres Strait) and where it has been predicted to have an expanded range in the future. So far, the use of an integrated modelling framework to complement vector control strategies has successfully prevented *Ae*. *albopictus* from establishing and spreading in Australia [51]. Such an approach can be used to diminish the range of other medically relevant endemic mosquito vectors of *Aedes* and *Culex* origin.

### The predictive impact of mechanistic species distribution modeling in Australia

Several MSDMs that have been used to project the future range of *Aedes* and *Culex* mosquito vectors in Australia under future climate and land-use change scenarios such as RCP 8.5, can be combined with SSDMs [84]. Examples of these MSDMs include CLIMEX [72], the container inhabiting mosquito simulation model (CIMSiM) and the Dengue simulation model (DENSiM) [86, 87, 101, 102], Niche Mapper ^TM^ (an ensemble of biophysical and evolutionary models) [73], and the Dynamic Mosquito Simulation Model (DyMSiM) [88]. These MSDMs have applications that extend beyond projecting a species future range. For instance, CIMSiM and DENSiM can and have been used to understand the processes that underlie the changing transmission patterns of arboviruses such as DENV by *Ae. aegypti* [72, 85–87, 101]. This application could be extended to endemic *Aedes* and *Culex* mosquito vectors that predominantly transmit RRV and BFV in Australia. Examples of vectors that commonly transmit RRV and BFV in Australia include *Ae. vigilax* and *Cx. annulirostris*. These vectors spread RRV and BFV to more than 5000 people each year [103]. But, if such biological and environmental information underlying the transmission of RRV and/or BFV by endemic mosquitoes is incorporated into integrated SDM frameworks, then such frameworks could significantly help minimise the spread of such viruses in Australia. In particular, the incorporation of integrated modelling frameworks may assist local authorities to implement integrated mosquito management programmes that will reduce exposure of the community to the pest and public health risks of mosquitoes. Strategic responses to the outcomes of modelling may include enhanced mosquito surveillance, implementation of site-species integrated mosquito control, or targeted community education programmes [51].

## Conclusions

This review has set out to demonstrate that utilising an integrated species distribution modelling framework consisting of both correlative and more mechanistic, process-based mosquito representations with climate and land-use change data is a useful and rigorous methodology to decipher the effect of changing environmental variables on future endemic or exotic mosquito-vector distributions. Such an integrated modelling framework has rarely been utilised to forecast the impacts of climate and land-use changes on mosquito habitat suitability most probably because of the paucity of literature and illustrations on how to do so. Combined use of SSDMs with MSDMs can have many advantageous but also some challenging outcomes such as divergent results. The most important advantageous outcomes include more robust, realistic and reliable predictions of species future range shifts or distributions, as well as an appreciation of the environmental factors and processes that greatly influence changes in species spread. Advantageous outcomes can be used to guide and improve the decision-making process and impact of future mosquito-vector control programmes implemented before, during and after arbovirus outbreaks. This is particularly important because Australia faces an ongoing challenge with arboviral diseases, and many of these diseases are either untreatable, lethal, and/or are transmitted by multiple endemic and exotic mosquito vectors whose range is impacted by climate and land-use changes.

## Data Availability

Not applicable.

## Abbreviations

BFV: Barmah Forest virus
CHIKV: Chikungunya virus
DENV: Dengue virus
RRV: Ross River virus
KUNV: Kunjin virus
JEV: Japanese encephalitis virus
JSDM: Joint species distribution model
MSDM: Mechanistic species distribution model
MVEV: Murray Valley encephalitis virus
SDM: Species distribution model
SSDM: Statistical species distribution model
ZIKV: Zika virus
